# The Geras virtual frailty rehabilitation program to build resilience in older adults with frailty during COVID-19: a randomized feasibility trial

**DOI:** 10.1186/s40814-023-01346-7

**Published:** 2023-07-17

**Authors:** Chinenye Okpara, George Ioannidis, Lehana Thabane, Jonathan Derrick Adachi, Alexander Rabinovich, Patricia Hewston, Justin Lee, Caitlin McArthur, Courtney Kennedy, Tricia Woo, Pauline Boulos, Raja Bobba, Mimi Wang, Samuel Thrall, Derelie Mangin, Sharon Marr, David Armstrong, Christopher Patterson, Steven Bray, Kerstin de Wit, Shyam Maharaj, Brian Misiaszek, Jessica Belgrave Sookhoo, Karen Thompson, Alexandra Papaioannou

**Affiliations:** 1 Department of Health Research Methodology, Evidence and Impact, McMaster University, Hamilton, ON Canada; 2 Geras Centre for Aging Research, Hamilton Health Sciences, Hamilton, ON Canada; 3 Department of Medicine, McMaster University, Hamilton, ON Canada; 4 The Research Institute of St Joseph’s Healthcare, Hamilton, ON Canada; 5 Faculty of Health Sciences, University of Johannesburg, Johannesburg, South Africa; 6 Department of Surgery, McMaster University, Hamilton, ON Canada; 7 School of Physiotherapy, Dalhousie University, Halifax, NS Canada; 8 Department of Family Medicine, McMaster University, Hamilton, ON Canada; 9 Department of Medicine, University of Toronto, Hamilton, ON Canada; 10 Department of Kinesiology, McMaster University, Hamilton, ON Canada; 11 Department of Emergency Medicine, Queen’s University, Kingston, ON Canada

**Keywords:** Older adults, Frailty, Feasibility studies, COVID-19, Virtual rehabilitation

## Abstract

**Background:**

The Coronavirus (COVID-19) pandemic has exacerbated the risk for poor physical and mental health outcomes among vulnerable older adults. Multicomponent interventions could potentially prevent or reduce the risk of becoming frail; however, there is limited evidence about utilizing alternative modes of delivery where access to in-person care may be challenging. This randomized feasibility trial aimed to understand how a multicomponent rehabilitation program can be delivered remotely to vulnerable older adults with frailty during the pandemic.

**Methods:**

Participants were randomized to either a multimodal or socialization arm. Over a 12-week intervention period, the multimodal group received virtual care at home, which included twice-weekly exercise in small group physiotherapy-led live-streamed sessions, nutrition counselling and protein supplementation, medication consultation via a videoconference app, and once-weekly phone calls from student volunteers, while the socialization group received only once-weekly phone calls from the volunteers. The RE-AIM (Reach, Effectiveness, Adoption, Implementation and Maintenance) framework was used to evaluate the feasibility of the program. The main clinical outcomes were change in the 5-times sit-to-stand test (5 × STS) and Depression, Anxiety and Stress Scale (DASS-21) scores. The feasibility outcomes were analyzed using descriptive statistics and expressed as frequencies and mean percent with corresponding confidence intervals (CI). Analysis of covariance (ANCOVA) was used for the effectiveness component.

**Results:**

The program enrolled 33% (*n* = 72) of referrals to the study (*n* = 220), of whom 70 were randomized. Adoption rates from different referral sources were community self-referrals (60%), community organizations (33%), and healthcare providers (25%). At the provider level, implementation rates varied from 75 to 100% for different aspects of program delivery. Participant’s adherence levels included virtual exercise sessions 81% (95% *CI*: 75–88%), home-based exercise 50% (95% *CI*: 38–62%), protein supplements consumption 68% (95% *CI*: 55–80%), and medication optimization 38% (95% *CI*: 21–59%). Most participants (85%) were satisfied with the program. There were no significant changes in clinical outcomes between the two arms.

**Conclusion:**

The GERAS virtual frailty rehabilitation study for community-dwelling older adults living with frailty was feasible in terms of reach of participants, adoption across referral settings, adherence to implementation, and participant’s intention to maintain the program. This program could be feasibly delivered to improve access to socially isolated older adults where barriers to in-person participation exist. However, trials with larger samples and longer follow-up are required to demonstrate effectiveness and sustained behavior change.

**Trial registration:**

ClinicalTrials.gov NCT04500366. Registered August 5, 2020, https://clinicaltrials.gov/ct2/show/NCT04500366

## Key messages regarding feasibility

- There is limited evidence on how multicomponent interventions to address frailty can be delivered remotely to older adults where there are health-related, geographical, or logistical barriers to access.
- We found that the Geras virtual multicomponent frailty rehabilitation program was feasible with respect to the reach of participants, adoption across referral settings, adherence to implementation protocol, and intention to maintain from participant’s perspective.
- A large-scale trial with longer follow-up is required to provide evidence of effectiveness and sustained behavioral change. Future trials should consider the potential for differences in feasibility of implementation in non-COVID-19 context, recruiting from multiple sources using different strategies for a wider reach of participants, providing devices for participation and optimal training of participants on how to navigate technology, and designing effective strategies to improve adherence to unsupervised home exercises, participant’s implementation of medication review recommendation, and retention of volunteers.

## Introduction

Frailty can be one of the challenging consequences of aging and is characterized by a decline in reserve and function across multiple body systems [1, 2]. In Canada, approximately 1.5 million older adults are frail, and this estimate is predicted to increase to more than 2 million within the next 10 years as the population ages [3]. Older adults living with frailty account for a large proportion of users of rehabilitation programs and home care services [4, 5]. Their decreased capacity to resist the negative impact of stressors increases the risk of experiencing adverse health outcomes, with costs to health and social care [6].

The COVID-19 pandemic presents a major stressor to vulnerable older adults. This population had the highest infection risk, illness severity, and case fatality [7]; consequently, they received the strictest public health preventive measures. Emerging evidence on the impact of the pandemic on older adults suggests decreased physical activity [8–11], increased sedentary behavior [12, 13], poor mental health [9, 10, 13, 14], and increased incidence of frailty [15, 16]. There are also indications of a negative impact on the nutritional behavior of this population with increased risk of undernutrition or overnutrition [17]. These factors could potentially exacerbate the risk of adverse consequences on their overall health and well-being. Therefore, interventions are critically needed to build resilience, preserve functional abilities, prevent frailty, and reverse or slow decline in older adults isolated at home.

International guidelines recommend the use of multidimensional rehabilitation approach including exercise, protein-calories supplementation, reduction of polypharmacy, and vitamin D3 supplementation to address frailty [18, 19]. Rehabilitation interventions are essential for building resilience, preserving functional capacity, and supporting recovery [20, 21]. Most trials on multicomponent frailty interventions were implemented before the COVID-19 pandemic [22, 23]. These trials were either conducted in-person or were hybrid (including in-person and virtual delivery or assessement) [24]. Face-to-face programs were not feasible with the early pandemic restrictions, thus necessitating innovative models of care that could be delivered remotely and safely.

Virtual rehabilitation offers a potentially viable alternative [24]; however, the evidence is sparse [25, 26]. Only 7% of the included studies in a recent scoping review of digital interventions investigated rehabilitation interventions [25]. While a recent meta-analysis found small positive effects on physical function and quality of life, the authors noted that there were insufficient details on implementation factors that could influence intervention outcomes [26]. Now that virtual care use is increasing [27–29], studies on virtual rehabilitation are needed to understand how best to deliver this service to older adults living with frailty. This evidence will contribute to improving equitable access to care, where there are barriers to participation in in-person programs. Our study reports the feasibility of a virtual multicomponent frailty rehabilitation program which was designed to build resilience among seniors living with frailty during the COVID-19 pandemic compared with a socialization-only intervention.

## Methods

### Study design, participants, and setting

This study was reported in accordance with the CONSORT extension for pilot and feasibility studies [30]. The Geras virtual multicomponent frailty rehabilitation study was a parallel group randomized controlled feasibility trial among community-dwelling older adults aged 65 years and above. The study was conducted between August 2020 and November 2021 during the peak of the pandemic in Canada and ended after the last cohort of participant completed the intervention. Participants were recruited from three referral sources: (1) healthcare providers, (2) community organizations, and (3) self-referrals from the community through advertising. Clinicians at the referral sources identified potentially eligible participants opportunistically during consultation using a clinical pre-screening checklist. The patients were asked for their permission to share their names, contact information (phone and email), caregiver information (name and phone number), and the pre-screening information with the Geras research team and for a member of the study team to collect their pre-screening information and contact them. Patients who consented were formally assessed for eligibility by the research team. For community organizations, potential participants were recruited pre-pandemic for the original study at the centers run by the organizations. They were referred by the center coordinators and had given permission to be contacted by the study team. When the study was adapted to virtual delivery, they were recontacted for consent, and those who consented to participate were assessed for eligibility. Self-referrals were interested individuals in the community who contacted the research staff by themselves through emails or phone call with the contact information provided on advertised materials. To be eligible for the study, participants had to (a) have a clinical frailty scale score of 4 to 6 indicating mild to moderate level of frailty [1], (b) ambulate independently with or without walking aid, and (c) have no other physical limitations to exercise evident by average resting heart rate of 50–100 bpm and average resting blood pressure ≤ 160/90 mmHg or for self-referrals have a clearance to exercise from their family physician. They were excluded if they (a) could not speak or understand English or had no caregiver for translation, (b) had difficulty following two-step instructions (assessed by asking if they could do that in a group exercise), (c) were receiving palliative care, (d) had unstable angina or heart failure, (e) would be unavailable for more than 20% of the duration of the study due to travel plans, and (f) were currently involved in a group exercise program. Potential participants were mailed study information document after an initial telephone contact to confirm interest. This was followed by an eligibility screening and consent visit for interested participants over the phone or via Zoom for Healthcare. Given that the study was completely virtually, verbal informed consent was obtained from participants following an in-depth discussion of study details with each person, which was then documented on a consent process form prior to participation in the study. Research assistants enrolled participants in cohorts of 10 and then randomly allocated to either the multimodal or socialization study arm with a 1:1 ratio based on a computer-generated block randomization sequence generated. Only the researcher who was not involved in the study had access to the computer-generated allocation list. Outcome assessors, analysts, and investigators were blinded to the participant group assignments. It was not possible to blind other study intervention personnel and the participants due to the nature of the intervention. Ethical approval for this study was obtained from the Hamilton Integrated Research Ethics Board (HiREB).

### Intervention development

The virtual frailty rehabilitation was originally designed as an in-person multicomponent community-based model of care to manage frailty which was adapted to a virtual delivery during the height of the COVID-19 pandemic. The modifications were based on existing evidence and discussions with stakeholders, including a team of researchers and healthcare providers to identify relevant and practicable solutions within the COVID-19 context. The exercises were informed by a systematic review on exercise interventions for frail older adults [31] and a meta-analysis on fall prevention in older adults [32]. The studies suggest that a combination of strength and endurance training performed at a moderate weekly frequency may improve muscle hypertrophy, strength, and power in frail older adults [31]. In addition, exercise performed for a minimum of 180 min/week with a high challenge to balance provides the greatest benefits for fall prevention [32]. The nutrition component aligned with recommendation for protein supplementation in older adults with frailty to enhance the gains of physical exercise [19]. Medication review was based on evidence that improving the appropriate use of polypharmacy in older adults can be obtained using the Beers’ criteria and Screening Tool of Older Person’s Prescription (STOPP)/Screening Tool to Alert to Right Treatment (START) [33]. The socialization component was initially designed as a group-based social engagement for better mental and physical health [34] but was modified due to the prevailing social and physical distancing measures during the pandemic.

### Intervention description

#### Multimodal arm

Participants randomized to the multimodal group received an intervention package comprising of exercise, medication support, nutrition, and socialization for 12 weeks. The intervention components are reported in accordance to the TIDieR guidelines [35].

#### Physical exercise

One-hour-long small group live exercise sessions delivered virtually via Zoom for Healthcare to participants at their homes were conducted twice weekly per cohort of participants assigned to the multimodal arm. The exercise classes were facilitated by physiotherapists with a participant-physiotherapist ratio of 5:1 per class. The physiotherapists were trained via videoconferencing by a physiotherapist co-investigator with expertise in exercise and rehabilitation for frail older adults and were provided with a manual developed by the expert. The exercises comprised of functional movements to build strength and balance and followed a sequence of 5-min warm-up exercises, 10-min aerobic activities, 20-min functional strength exercise, 20-min balance training, and 5-min warm down and stretching exercises. All multimodal participants were provided with an exercise safety sheet that included tips for exercise preparation, materials required, and safety considerations. They were also given safety cues for correct posture, body position, and equipment safety during the virtual sessions. Participants were allocated time at the beginning of the class to report any concerns or injuries. They were given additional tailored home-based exercises to be performed for at least 1 h in order to achieve the minimum 3 h/week of exercise required for fall prevention [32]. The home-based exercises were developed from what was taught during the virtual exercise sessions and were routinely reviewed for safety and level of challenge appropriateness by the study physiotherapists.

#### Nutrition support and protein supplementation

For the nutrition component, multimodal participants had an individualized virtual nutrition assessment and coaching session on how to improve their nutrition with a research assistant who was trained by a dietitian. The nutrition counselling was developed with the guidance of geriatric nutrition experts. In addition, the participants received oral protein supplements and protein intake adherence tracking logs via contactless delivery. Participants were either provided commercially available protein drink or powder depending on their preference, and those who were diabetic were given suitable alternatives. During the nutrition counselling and review session, participants who had concerns about the protein supplement were recommended to speak with their family physician about it. The nutrition supplement contained 360 cal and 14 g of protein per serving to be taken daily with a meal or within 3 h of exercise.

#### Medication review consultation

The medication support intervention included a one-on-one virtual visit with the trained study pharmacist. The visit involved the review of participants medical record and medication list, followed by providing recommendations to their family physician or pharmacist where necessary. Optimization of medications was conducted using STOPP/START [36] and Beers criteria [37]. Participants were asked to review the recommendations with their primary care provider. A follow-up check-in occurred at their 12-week appointment to determine whether the medication recommendations were implemented.

Participants in the multimodal arm received the same socialization intervention as those in the socialization arm described below. All study personnel — blinded assessors, nutrition counsellor, pharmacists, physiotherapist, and social call volunteers, were trained on study protocols by the research team before study implementation and were provided with relevant study materials.

#### Socialization arm

#### Social calls

The socialization component involved a once-weekly phone call from trained volunteers to participants in both socialization and multimodal arms to mitigate the impact of social isolation during the pandemic. The volunteers consisted of undergraduate, graduate, and medical school students. They were each assigned to a maximum of two participants. The conversations were unstructured; however, volunteers were provided with prompts that covered topics related to COVID-19, wellness, and life experiences including family, hobbies, and work. All volunteers received an hour-long synchronous and asynchronous training on communication with older adults by a study research assistant.

At the end of the study, participants in the socialization arm were offered the opportunity to participate in a 2-week long virtual exercise program post-intervention period.

### Technology use

Persons who indicated interest in the study but did not have devices or internet connection were given iPads and internet service. Participants were oriented to the use of the devices during a brief phone conversation with study research staff and were provided with tip sheets on how to navigate the devices. Technical challenges regarding connectivity, audio, or visuals were addressed earlier in the study during baseline assessments or in the first week of the exercise classes.

### Sample size estimation

Enrolment rateThe sample size is based on the implementation feasibility success threshold of 75% adherence to intervention components. Considering a 10% dropout rate, we needed a sample size of 70 participants (35 participants in the multimodal intervention group and 35 participants in the socialization group) to produce a two-sided 95% confidence interval with a width equal to ± 11%. This sample size was large enough to provide useful information regarding feasibility that will inform a larger multicenter trial. The sample size calculation was conducted using PASS software (Kaysville, Utah).

### Evaluation and analysis of program feasibility

The RE-AIM framework [33] was used to evaluate the feasibility of the program. The framework considers five elements (Reach, Effectiveness, Adoption, Implementation and Maintenance) that could influence the implementation success and impact of a program [38]. RE-AIM has been used in the evaluation of the feasibility and implementation of similar health programs [39–41]. Given the unique COVID-19 implementation context and novelty of the intervention, the framework allows for the use of multiple indicators to broadly assess and understand the factors that could impact future study outcomes. Table 1 outlines how we applied the RE-AIM framework in this study including component definitions, outcome measures, and criteria for success. Briefly, *Reach* was defined as recruitment of target population. It was assessed by enrollment rate (percentage of all referrals enrolled in the study) and by the examination of participant demographics. The feasibility threshold for this component was set at an enrolment rate of ≥ 10%, derived from previous studies on digital intervention in frail older adults [42, 43]. *Effectiveness* was assessed by comparison of physical function using the five times sit-to-stand test (5XSST) [44], psychological distress using the Depression, Anxiety and Stress Scale (DASS-21) [45] and adverse events between the multimodal and socialization arm. *Adoption* was measured by the percentage of participants enrolled from each referral source. Success for this domain was defined as having each referral sources contributing ≥ 10% of enrolled participants for representativeness of settings. *Implementation* was assessed by adherence to each component of the intervention either at participant or provider (i.e., study research team) level. For the exercise component, implementation was evaluated by the percentage of virtual exercise sessions attended out of total number of sessions and percentage of home exercises completed out of total number expected. For the nutrition element, the measures were based on percentage of participants whose protein shipment was successfully delivered at provider level and percentage of protein supplements consumed out of total supplements at participant level. We assessed medication review as percentage of participants who received medication consultation from the study pharmacists at provider level and percentage of participants who implemented medication recommendation at participant level. The socialization component was measured by the percentage of calls made per participant out of the total calls at provider level. Success was defined as achieving ≥ 75% adherence and ≥ 75% implementation for participants and providers, respectively. *Maintenance* was defined as intention to sustain the intervention. Since the study duration was short, we were unable to measure actual sustainability or long-term effects of the intervention; as such, proxy measures based on program satisfaction survey were used. This was assessed by the percentage of participants who would recommend the program (≥ 7/10 rating on the question how likely are you to recommend the program?) and percentage of participants who reported that the program met their expectations. Maintenance was considered a success if ≥ 75% participants were satisfied with or would recommend the program.

**Table 1:** RE-AIM assessment of program feasibility

			**Results**	
**RE-AIM component**	**Outcome measure**	**Criteria for success**	**Outcome**	**% (95% CI)**
**Reach**	**Recruitment**			
Recruitment of target population	Proportion of persons who were enrolled out of all referrals (participation rate)	10% of all referrals enrolled in the study	Enrolment rate	72 (33)*
	Assessment of participants’ characteristics			
**Effectiveness**				
Positive and adverse effect of intervention	5XSST	**-**	Please see Table 3	
	DASS 21	**-**		
	Adverse events	**-**		
**Adoption**	**Referral sources and settings**			
Representativeness of settings	Proportion of participants enrolled from different sources	≥10% of participants from each source enrolled in the study	**Referral sources**	
			Health provider	42 (25)*
			Self-referral	28 (60)*
			Community organization	2 (33)*
**Implementation**	**Adherence to intervention**			
Successful delivery of intervention, fidelity, and modifications to interventions	**Social calls**	**Social calls**	**Social calls**	
	Percentage of weekly calls made per participant out of the total calls	75% of the participants received all weekly calls	Percentage calls volunteers made per participant	78 (71 – 84)
	**Exercise**	**Exercise**	**Exercise**	
	Percentage of virtual exercise sessions attended	Group: ≥75% of class attendance	Average virtual exercise sessions attended	81 (75 – 88)
	Percentage of home exercises completed	Home: ≥75% of home exercise completion	Average home-based exercises completed	50 (38 – 62)
	**Nutrition**	**Nutrition**	**Nutrition**	
	Percentage of participants who received protein supplements	≥75% of the participants received their protein supplements	Received protein supplements	97 (80 – 100)
	Percentage of protein supplements consumed	≥75% of the participants had daily protein supplements	Average daily protein supplements consumption	68 (55 – 80)
	**Medication review**	**Medication review**	**Medication review**	
	Percentage of participants who received medication review	≥75% received a medication review consultation	Received medication review	33 (100)*
	Percentage of participants who implemented recommendations	≥75% implemented the recommendations	Implemented recommendation	38 (21 – 59)
	**Outcome assessment**			
	Average time to complete assessment of outcomes			
	**Project devices**			
	Percentage of participants who required project iPad device			
	**Intervention personnel**			
	Number of volunteers who were trained and dropped out			
**Maintenance**	**Satisfaction survey**			
Intention to sustain intervention	Percentage of participants who completed end-of-study surveys			
	Percentage of participants who were satisfied with the program	≥75% of participants would recommend the program (≥7/10 rating)	Satisfied with the program	86 (71 – 94)
	Percentage of participants who will recommend the program	≥75% of participants are satisfied with the program (that is program met their expectations)	Would recommend the program	76 (61 – 87)

*DASS-21*, Depression Anxiety and Stress Scale, *5XSST* Five times sit-to-stand; ≥ , greater than or equal to; *count (percentage)

### Data collection

Data collection was conducted virtually via Zoom for Healthcare or the telehealth through Clinicmaster, at baseline and 12 weeks of follow-up by blinded assessors with rehabilitation training. Outcome assessors were trained and observed by research staff to standardize the assessments and ensure it was done appropriately.

### Clinical outcome measures

#### Physical function

Five times sit-to-stand was used to assess lower limb strength [44]. It is a feasible, reliable, and valid measure for mobility and falls [46] with moderate sensitivity to change over time [45].

#### Psychological distress

Depression, Anxiety and Stress Scale (DASS-21) is a short version of the DASS-42 used to assess negative emotional status [47]. It has good psychometric properties and wide applicability to different populations [48].

#### Other measures

These include participants’ baseline demographics, adherence to intervention, and satisfaction survey. Participant’s attendance at each virtual exercise session was recorded by study physiotherapists, and participants tracked their adherence to prescribed home-based exercise on an exercise log. Study personnel performed biweekly phone check-ins to monitor adverse events and track protein supplementation distribution and use. Adverse events collected include exercise related (fall, fracture, pain with exercise, dizziness, muscle strain, sprain, respiratory, and cardiac adverse events) and nutrition related (constipation, diarrhea, upset stomach, severe weight loss and gain, and renal adverse events). Participants who had medication recommendations were reminded by study staff to review the recommendations with their primary care provider. Information regarding the implementation of recommendations were collected during the last two phone check-ins with the participants. Attendance and duration of the socialization phone calls were recorded by the volunteers. To obtain feedback about the program, participants and study implementation personnel completed an online satisfaction survey anonymously.

### Statistical analysis

Participant’s baseline characteristics were described as means with standard deviation and frequencies with percentages for continuous and categorical variables respectively. The feasibility outcomes were analyzed using descriptive statistics and expressed as frequencies, mean percent with corresponding confidence intervals (CI). Effect of the intervention was assessed using between-group analysis of covariance (ANCOVA) adjusting for baseline scores and was based on intention-to-treat (ITT) principle. Multiple imputation using chained equation was used to account for missing values assuming the data were missing at random. A sensitivity analysis based on per-protocol (PP) cohort, that is, participants who completed the trial and who had complete data, was performed to assess if there were any difference in effects for those who completed the trial. Results are presented as pre- and postintervention means, adjusted mean differences with associated 95% confidence interval (CI). All analyses were performed using Stata version 17 (StataCorp, College Station, TX, USA).

## Results

### Participant characteristics

**Fig. 1:**
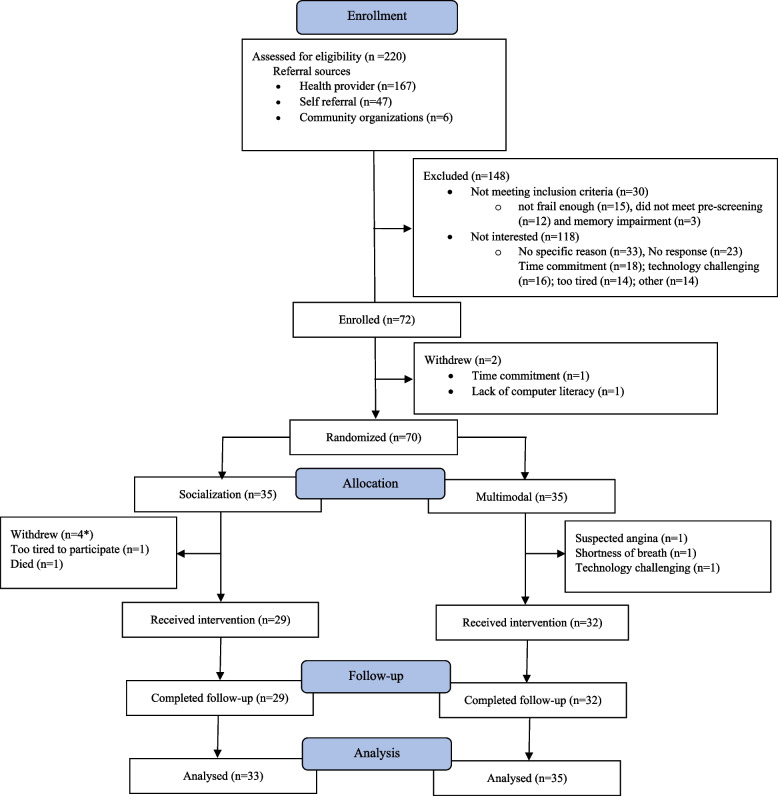
CONSORT flow chart of participants. *Data removed as requested by participant (*n*=2)

The study enrolled participants between September 2020 and July 2021. Table 2 presents the baseline characteristics of participants by study arm. Of the 70 randomized participants, 67 had a baseline assessment, 32 in the socialization, and 35 in the multimodal study arms. The average age of the participants was 77.3 (*SD*: 6.4), of whom 12 (18%) were frail, 29 (58%) were prefrail, and 16 (24%) were non-frail (based on a frailty index categorization[49]). The majority of participants were females 53 (77%), had college or university education 46 (68%), and lived with others 41 (60%). The mean time to complete 5XSST was 15.6 (*SD*: 7.0) s, and mean frailty index score was 0.26 (0.09). The mean scores for depression, anxiety and stress were 6.7 (*SD*: 6.6), 5.2 (*SD*: 4.8), and 8.1 (*SD*: 6.8), respectively. Figure 1 shows the CONSORT flowchart of study participants.

**Table 2:** Baseline characteristics of participants

**Characteristics**	Total *n* = 67	Socialization arm *n* = 32	Multimodal arm *n* = 35
Age, mean (SD)	77.3 (6.5)	76.4 (5.8)	78.2 (7.0)
Age *n* (%)			
65–74	24 (35.8)	13 (40.6)	11 (31.4)
75–84	33 (49.3)	16 (50.0)	17 (48.6)
85 +	10 (14.9)	3 (9.4)	7 (20.0)
Gender *n* (%)			
Male	15 (22.4)	9 (28.1)	6 (17.1)
Female	52 (77.6)	23 (71.9)	29 (82.9)
Living arrangement *n* (%)			
Lives alone	27 (39.7)	11 (33.3)	16 (45.7)
Lives with others	41 (60.3)	22 (66.7)	19 (54.3)
Educational level *n* (%)			
≤ 12th grade	10 (14.7)	2 (6.2)	8 (22.9)
High school	12 (17.7)	6 (18.8)	6 (17.1)
College	23 (33.8)	11 (34.4)	12 (34.3)
University	22 (33.8)	13 (40.6)	9 (25.7)
Smoking status *n* (%)			
Current	2 (3.0)	0 (0.0)	2 (5.7)
Former	15 (22.4)	5 (15.6)	10 (28.6)
Never	50 (74.6)	27 (84.4)	23 (65.7)
Frailty status			
Non-frail	16 (23.9)	9 (28.1)	7 (20.0)
Prefrail	39 (58.2)	16 (50.0)	23 (65.7)
Frail	12 (17.9)	7 (21.9)	5 (14.3)
Falls in the past year *n* (%)			
No	32 (47.8)	16 (50.0)	16 (45.7)
Yes	35 (52.2)	16 (50.0)	19 (54.3)
Walking aid use *n* (%)			
No	34 (50.8)	16 (50.0)	18 (51.4)
Yes	33 (49.2)	16 (50.0)	17 (48.6)
Previous fractures^1^ *n* (%)			
No	39 (58.2)	16 (50.0)	23 (58.2)
Yes	28 (41.8)	16 (50.0)	12 (41.8)
5XSST mean (SD)	15.6 (7.0)	13.5 (4.9)	17.5 (8.0)
DASS-21 depression, mean (SD)	6.7 (6.6)	7.4 (5.7)	6.2 (7.3)
DASS-21 anxiety, mean (SD)	5.2 (4.8)	5.4 (4.1)	5.0 (5.5)
DASS-21 stress, mean (SD)	8.1 (6.8)	9.6 (6.8)	6.7 (6.6)
Frailty index, mean (SD)	0.26 (0.09)	0.25 (0.10)	0.27 (0.08)
EQ-5D-5L index, mean (SD)	0.77 (0.15)	0.78 (0.12)	0.76 (0.17)

^1^Fracture since the age of 50 years; *n*, number of participants; %, percentage; *SD*, standard deviation; *DASS-21*, Depression, Anxiety and Stress Scale; *5XSST*, five times sit-to-stand; EQ-5D-5L, measure for health-related quality of life

### Feasibility

#### Reach

We had a total of 345 referrals, of whom approximately 36% (*n* = 125) were waitlisted for another study after the required sample size was attained. The remaining 220 referrals were assessed for eligibility; of these, 72 were enrolled, representing a reach of 32.7%. The major reasons for exclusion were as follows: refusals to participate without any specific reason 33 (22%), not meeting inclusion criteria 30 (20%), and no response when contacted by study personnel 23 (16%).

#### Effectiveness

As shown in Table 3, there was no statistically significant difference between the groups in either the intention-to-treat and the per-protocol analyses for time to complete 5XSST (ITT: *aMD* =  − 0.59, *CI*: − 3.51–2.33; PP: *aMD* =  − 1.28, *CI*: − 4.01–1.45), depression (ITT: *aMD* =  − 0.66, *CI*: − 3.62–2.29; PP: *aMD* =  − 0.76, *CI*: − 3.48–1.97), anxiety (ITT: *aMD* = 0.49, *CI*: − 1.41–2.40; PP: *aMD* =  − 0.08, *CI*: − 1.63–1.79), and stress (ITT: *aMD* = 0.380, *CI*: − 3.29–4.05; PP: *aMD* =  − 0.0559, *CI*: − 4.08–2.96). There were 45 exercise or nutrition-related adverse events reported with no difference between the multimodal and socialization arm (25 (78%) vs 20 (69%)). The adverse events were largely exercise related 43 (96%), and most were falls 23 (54%). Only 2 (4%) nutrition-related adverse events were reported. Both study arms had about the same number of falls [multimodal 12 (36%) vs socialization arm 11 (38%)] over the course of the study. One death occurred in the socialization arm before baseline assessment and intervention started.

**Table 3:** Clinical outcome analyses

	**Socialization**		**Multimodal**			
	**Pre**	**Post**	**Pre**	**Post**	**aMD**	**95% *CI***
**ITT**						
5XSST	13.7	14.0	17.8	15.3	− 0.59	− 3.507–2.326
Depression	7.4	7.4	6.2	6.0	− 0.66	− 3.615–2.285
Anxiety	5.4	4.9	5.0	5.1	0.49	− 1.411–2.398
Stress	9.6	9.2	6.7	7.6	0.38	− 3.285–4.047
**PP**						
5XSST	13.6	14.0	16.3	14.6	− 1.28	− 4.007–1.454
Depression	7.7	7.7	5.4	5.7	− 0.76	− 3.484–1.969
Anxiety	5.4	5.0	5.4	5.1	0.08	− 1.634–1.794
Stress	10.1	9.8	6.5	7.1	− 0.56	− 4.076–2.958

*ITT* Intention to treat, *pp* Per protocol, *aMD* Mean difference between socialization and multimodal arm adjusted for baseline score, *CI* Confidence interval, *5XSST* Five times sit-to-stand

#### Adoption

Of the 220 people referred for enrollment, 167 were from health providers of whom 42 (25%) were enrolled in the study. Forty-seven people contacted the study themselves, of whom 28 (60%) enrolled. Six people were referred from community organizations of whom 2 (33%) enrolled in the study. Of the 72 study participants enrolled in the study, 42 (58%) were referred by health providers, 28 (39%) were self-referrals, and 2 (3%) were from community organizations.

#### Implementation

Twenty-four virtual exercise sessions were conducted per cohort of participants in the multimodal group. The average class attendance was 81% (95% *CI*: 75–88%), and adherence to the home-based exercise was 50% (95% *CI*: 38–62%). No difference was observed in adherence between participants who had their own device and those who were given study device for participation (81% vs 82%). There were no major deviations from the protocol. For the socialization component, 78% (95% *CI*: 71–84%) of expected calls were made, and the mean call duration was 21 min (*SD*: 10.4) in both groups. The major challenges were the organization and attrition of the pool of trained student volunteers for the social calls. A total of 47 volunteers was trained, of whom 12 (26%) dropped out before assignments. In some instances, when volunteers dropped out or were unavailable for the calls, another volunteer was assigned to the participant. Regarding the nutrition supplementation, 97% (95% *CI*: 80–100%) of the participants received the first shipment of protein supplements, while 27 (84%) received all their protein supplements. The 5 participants (16%) who did not receive all the protein supplements opted out for personal reasons including feelings of self-sufficiency with nutrition. The average consumption rate for the protein supplements over the 12-week intervention period was 68% (95% *CI*: 55–80%). All 33 intervention participants had a consultation with the study pharmacist representing 100% implementation. Twenty-six out of 33 (79%) had a recommendation to optimize their medication, and only 38% (95% *CI*: 21–59%) of these participants implemented the recommendations. Among those who had a recommendation, 13 (57%) reviewed the recommendations with their family physician but did not implement them.

#### Maintenance intention

Forty-two (60%) of the participants, 30 (71%) multimodal, and 12 (29%) socialization arm responded to the end-of-study satisfaction survey. Among them, 85% (95% *CI*: 71–94%) were satisfied with the program, and this differed between the two groups (multimodal 28 (93%) vs socialization 8 (67%)). Thirty-two 76% (95% *CI*: 61–87%) would recommend it with no difference between the multimodal 24 (80%) and socialization 8 (67%) arm. Of the 29 participants in the socialization arm who received the intervention, 19 (66%) engaged in the post-study exercise intervention. The reasons for nonparticipation include technology challenge, vacation, and no interest.

The average time to complete outcome assessments virtually per participant was 51 (*SD*: 9.0) min. There were no adverse events reported during the assessments. Ten participants (14%) were provided an iPad device for outcome assessment and the virtual exercise sessions. Of the 72 participants enrolled, 61 (85%) completed the study. Please see Table 1 for details of feasibility results.

## Discussion

The RE-AIM evaluation showed that the Geras virtual frailty rehabilitation program was feasible in terms of reach, adoption across different referral settings, adherence to implementation, and intention to maintain based on the predefined criteria for success. However, the pandemic context may have added a layer challenge to some aspects of the program including slow adoption in some settings and participants’ adherence to some intervention components. While these findings are promising, a larger trial with longer follow-up is required to determine effectiveness and sustained behavior change.

In terms of reach, the participation rate was satisfactory and comparable to similar studies conducted in-person or hybrid (online and face to face) before the pandemic [50–52]. Enrollment of eligible participants was slow for the most part of the recruitment phase but increased dramatically towards the end. The major facilitator to recruitment was the dissemination of stories of participants enrolled earlier in the study through print and broadcast media. Conversely, the use of targeted social media advertising yielded lower response rates. Emerging evidence suggests that online recruitment strategies could be effective [53, 54]; however, it may most likely benefit persons who are already familiar with and have access to these digital tools and platforms [55]. A combination of strategies may be better at reaching a wider range of potential participants including technology-familiar and naïve persons. To maximize representativeness and equity in the study, we extended eligibility criteria to include persons without access to a device for participation, and approximately 15% of enrolled participants did not have their own devices and internet service. Despite these efforts, there were fewer males, persons with limited education, and the oldest-old enrolled in the study. These population tend to have lower participation in digital health research [56–58]. As such, further studies are required to understand potential strategies to improve equitable participation among these underrepresented groups.

Given that the study was not powered to detect a difference in effects between groups, we cannot make conclusive interpretations about the effectiveness of the intervention. Although the 5XSST measure of physical function showed a meaningful clinical reduction of 2.5 s in time to completion in the multimodal group [45], the difference was not statistically significant when compared to the socialization arm and adjusted for baseline scores. Notwithstanding, faster times could translate to gains in functional independence and ability to perform activities of daily living [59]. For the measures of psychological well-being (depression, anxiety, and stress), the observed differences were either in the negative or positive direction for both multimodal and socialization arms but were not significant clinically important effects. A larger trial could provide definitive evidence on the benefits of virtual rehabilitation.

In terms of safety, there were no adverse events related to the intervention as both multimodal and socialization arms had about the same number of events. In addition, no falls occurred during the virtual exercise sessions, and no adverse events were reported during the virtual outcome assessment. This finding suggests that virtual rehabilitation for frail older adults could be safely implemented; however, more research is needed to demonstrate its safety as this has not been well-reported in the literature [26].

Adoption threshold was met for the three referral settings but was not as high for healthcare settings and community organizations. Decreased access to care [60], shifts to virtual care [28, 29], and fear of contracting the virus during hospital visits [61, 62] may have affected patients’ decisions to seek care as they would normally do in pre-pandemic times. Additionally, most patients who were seen during that period were very ill [61] and were probably too weak to participate in the study as indicated in the reasons for declining. We speculate that a combination of these pandemic-related factors could have contributed to the lower than anticipated enrolment rates recorded from healthcare settings. Similarly, the small number of persons reached and enrolled from community organizations could be attributed to the prevailing pandemic restrictions that hindered access to the pool of older adults, who hitherto attended in-person services at the seniors’ centers run by these organizations before the pandemic. However, it is possible that our targeted media recruitment strategy, which generated a high yield, may have captured some potential participants, who had been missed from this source through self-referrals from the community.

Implementation outcomes were promising for the delivery of all intervention components with adherence rates varying from 75 to 100% and no major modifications. Given the challenging context of implementation, the observed adherence to program delivery is encouraging for future virtual rehabilitation interventions. The virtual exercise class attendance rate was comparable to the adherence rates reported in systematic reviews of technology-based exercise programs in older adults [63, 64]. Being able to deliver exercise sessions virtually to 5 persons per physiotherapist per time is promising for rehabilitation care in Canada given the long wait times for home care services [65]. It is important to note that the virtual exercise adherence rate was similar between those who had their own devices and those who received study devices and internet service to participate in the program. This suggests that disadvantaged groups (persons without access to technology) could benefit from digital health research with same level of adherence as their counterparts even with the minimal training on the use of these devices that was provided by the study. Conversely, adherence rate to the structured homework exercise was not as optimal, which is consistent with existing research [66, 67]. This could result in decreased gains from the supervised physical therapy program [68] and decreased attainment of therapeutic goals [69], as the homework exercises were intended to complement the virtual exercise sessions for the fulfillment of the minimum exercise requirements for fall prevention [32]. More research is required to understand how to sustain behavior change among older adults in unsupervised exercise programs. Regarding the nutrition component, participants’ adherence to protein supplements was lower than rates reported in recent systematic reviews of nutrition interventions (68% vs > 90%) [70, 71]. It is important to note that the studies included in these reviews were conducted pre-pandemic times. It is possible that pandemic-induced circumstances not captured in this study may have influenced participants’ nutrition compliance [17]. Adherence to the medication review recommendations was especially poor. Participants reported having difficulties with booking appointments with their family physician to make the recommended changes to their prescriptions, after the consultation with the study pharmacist. The limited access to healthcare services during the pandemic [60] could have hindered the next step in the study’s medication optimization process. The reasons are not known for participants who consulted with their family physician but did not implement the study pharmacist’s medication review recommendations.

Overall, feedback about the program was largely positive with high ratings for satisfaction and recommendations by majority of the participants, suggesting that future virtual rehabilitation programs for vulnerable older adults may be well-received. However, it is important to note that these measures were used as a proxy for assessing intention to maintain, and as such may not reflect acceptance in a world without pandemic restrictions, where there are options for in-person programs. Maintenance would be more appropriately evaluated in a study with a longer duration and post-intervention follow-up period that includes both participants and providers perspectives [38, 72].

### Strengths and limitations

This feasibility study has several strengths. First, the inclusion criteria extended to disadvantaged groups who have no device for digital interventions, thus promoting equity in access to care. The study was purely virtual, as all study implementations including outcome assessment were done remotely. We used the RE-AIM framework which allowed for a comprehensive evaluation of all aspects of the program at both participant and provider levels. Despite these strengths, this study has some limitations to consider. Most data were based on self-reports, so there is possibility of underestimation or overestimation of measures. Our study lacked data on assessment of fidelity to delivery of the intervention components which is important for future trials. The effectiveness analyses were exploratory as our study was not sufficiently powered to detect statistical significance; therefore, the results should be interpreted with caution. We were not able to objectively assess the maintenance domain of the RE-AIM framework in this short-term feasibility study; as such, the finding may not reflect actual sustainability. Additionally, the majority of respondents were from the multimodal arm which could have systematically influenced the outcomes measured through the satisfaction survey.

## Conclusion

Our study demonstrates that a virtual rehabilitation program for socially isolated, community-dwelling older adults could be feasibly implemented. Considering that it was conducted in the early phases of COVID-19 pandemic when public health restrictions were in place, feasibility outcomes may be different post COVID-19, when there are no social and physical distancing restrictions. Notwithstanding, the virtual mode of rehabilitation presents a promising option that could complement in-person programs in the immediate and post-COVID 19 era, particularly, where access to these interventions may be challenging, for example, due to mobility impairments, shortage of services, and/or long wait times. However, larger definitive trials are required to provide evidence of effectiveness and sustained behavior change. These trials may need to consider recruiting from multiple sources using different strategies for a wider reach of participants. Also, continuously monitoring and adapting recruitment strategies as well as highlighting and sharing participants’ success stories to motivate potential participants and enhance the visibility and credibility of the study could increase the chances of reaching intended sample size. We suggest providing devices and optimal training on how to navigate the technology to potential participants who do not have access to these resources to promote access and equitable participation in digital health research. Future trials should consider strategies on how to improve adherence to unsupervised home exercises, optimize medication review process, and retain social call volunteers.

## Data Availability

The data are held by a third party and include potentially identifying patient information so cannot be shared publicly. However, the dataset is available upon request from the principal investigator Dr. Alexandra Papaiaonnou: papaioannou@hhsc.ca, Geras Centre for Aging Research, Hamilton, Health Sciences, Hamilton, ON, Canada.

## Abbreviations

COVID-19: Coronavirus disease
RE-AIM: Reach, Effectiveness, Adoption, Implementation, and Maintenance
5XSST: Five times sit-to-stand test
DASS: Depression, Anxiety and Stress Scale
ITT: Intention to treat
PP: Per protocol
aMD: Adjusted mean difference
SD: Standard deviation
ANCOVA: Analysis of covariance
CI: Confidence intervals
HiREB: Hamilton Integrated Research Ethics Board
STOPP/START: Screening Tool of Older Person’s Prescriptions and Screening Tool to Alert to Right Treatment
